# Engineering a redox-active interface for highly reversible aluminum anode-based practical all-solid-state lithium batteries with ultralow N/P ratio

**DOI:** 10.1039/d6sc03781j

**Published:** 2026-06-01

**Authors:** Jiawu Cui, Xiaohui Sun, Zhenxin Huang, Xianwei Wang, Zhen Wang, Zhanhui Jia, Chao Wu, Kang Yang, Yuping Wu, Wei Tang, Ya-Ling He

**Affiliations:** a School of Chemical Engineering and Technology, National Innovation Platform (Center) for Industry-Education In-tegration of Energy Storage Technology, State Key Laboratory of Fluorine & Nitrogen Chemicals, Xi'an Jiaotong University Xi'an 710049 P. R. China; b State Key Lab Space Power Sources, Shanghai Institute Space Power Sources Shanghai 200245 P. R. China; c School of Materials Science and Engineering, Xi'an Jiaotong University Xi'an 710049 P. R. China; d Top-Energy Digital Manufacturing Technologies (Xi'an) Co., Ltd No. 2090, Hangtuo Road Xi'an 710100 P. R. China; e School of Energy and Environment, Southeast University Nanjing 210096 P. R. China

## Abstract

Aluminum is a promising anode for all-solid-state lithium batteries (ASSLBs) owing to its high theoretical capacity (900 mAh g^−1^) and optimal lithiation potential. However, its practical viability with critical N/P ratio and high current density is plagued by mechanochemical failure, sluggish kinetics, and extremely low reversibility. Herein, we construct a redox-active interface (comprising Li_2_S, Li_*x*_P, *etc.*) on the Al anode *via* the electrochemical activation of a Li_5.4_PS_4.4_Cl_1.6_ sulfide electrolyte. This interphase concurrently accelerates Li^+^ transport and fortifies interfacial stability. Theoretical modeling establishes the Li^+^ binding energy difference (Δ*E*) as a critical descriptor for interfacial stability; a substantial Δ*E* strongly confines Li^+^ within the anode bulk, effectively preventing parasitic ion migration and interfacial degradation. Consequently, the engineered Al anode delivers near-theoretical capacity and exceptional reversibility. Strikingly, the ASSLBs sustain over 1000 cycles under practically demanding conditions: a low N/P ratio of ∼1.05, a high-loading cathode (30 mg cm^−2^), and a high current density of 7 mA cm^−2^, setting a new benchmark for practical operations. Coupled with the ultra-low cost of Al powder (3.77 USD per kg), this redox-interface strategy unlocks a highly viable pathway for cost-effective, high-energy-density ASSLBs.

## Abstract 2

The Al anode featuring an electrochemically formed redox interface delivers substantially improved electrochemical performance relative to a pristine Al foil electrode.

## Graphical abstract

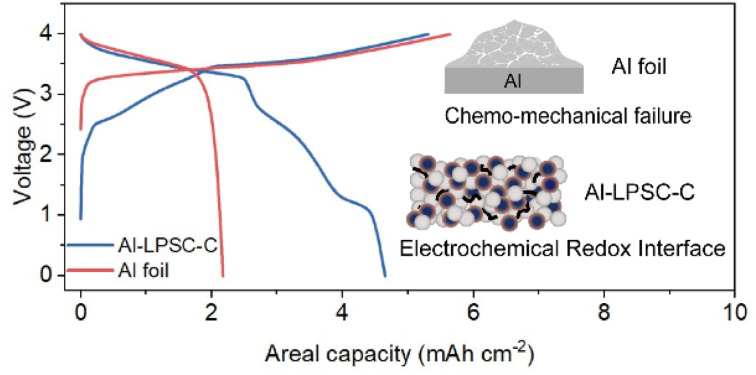

## Introduction

The rapid electrification of transportation has significantly increased the demand for high-energy-density and safe energy storage systems.^1,2^ Among the emerging solutions, all-solid-state lithium batteries (ASSLBs) employing sulfide solid-state electrolytes (SSEs) have attracted considerable attention as potential successors to conventional liquid-state lithium-ion batteries.^3–5^ This is largely due to the high ionic conductivity of sulfide electrolytes and their potential compatibility with high-capacity anode materials, collectively providing a pathway to overcome the intrinsic limitations of current energy storage technologies.^6–8^ Lithium metal anodes (LMAs) have long been regarded as the “Holy Grail” for maximizing battery energy density.^9,10^ However, their practical implementation in sulfide-based systems is hindered by well-known challenges: uncontrolled dendrite growth that can penetrate the electrolyte and cause short circuits, as well as thermodynamic instability at the Li/sulfide interface, which leads to continuous consumption of active lithium.^11–13^ As a result, it remains a significant challenge to design a lithium anode architecture that mitigates safety hazards and suppresses interfacial parasitic reactions while maintaining high specific capacity.^14,15^

In this context, aluminum (Al) has attracted considerable attention as a promising alternative anode material.^16^ Al offers a high theoretical volumetric capacity (∼8040 mAh cm^−3^ for Li_9_Al_4_), along with earth abundance and low cost.^17^ More importantly, the lithiation potential of Al (∼0.3 V *vs.* Li/Li^+^) is slightly higher than that of lithium metal plating, intrinsically reducing the risk of dendrite formation while maintaining a high operating voltage for Al anode-based full cells.^18,19^ Furthermore, ASSLBs employing pure Al-based anodes can achieve energy densities comparable to those of lithium metal-based systems, providing a potential solution to reconcile the intrinsic trade-off between safety and energy density.^20–22^

Despite these advantages, the application of pure Al-based anodes in sulfide-based ASSLBs is severely limited by chemo-mechanical failure.^20^ Lithiation induces drastic volume expansion (*e.g.*, ∼97% for the β-LiAl phase),^23^ generating substantial mechanical stress within the rigid solid-state cell. This expansion leads to anode particle pulverization, loss of electrical contact, and fracture of the solid electrolyte interphase (SEI), ultimately resulting in low initial coulombic efficiency (ICE < 80% for Li/Al alloy anode, ICE < 50% for pure Al anode) and rapid capacity fading.^24–26^ In addition, the intrinsically sluggish Li^+^ diffusion within the Al matrix restricts the feasibility of high-loading electrodes, thereby limiting the practical areal capacity.^20,27,28^ Consequently, achieving a highly reversible pure Al-based anode with fast ionic conduction and stable interfaces remains a critical bottleneck for the development of Al-based ASSLBs.

Herein, we report a highly reversible Al composite anode achieved by homogeneously blending pure Al powder (an exceptionally low-cost raw material at merely 3.77 USD per kg) with the sulfide SSE of Li_5.4_PS_4.4_Cl_1.6_ (LPSC). Unlike the conventional strategies in which SSEs solely act as ionic conductors, we demonstrate that LPSC within the Al anode/carbon matrix can be electrochemically actively to form a redox-active interphase between Al anode and LPSC SSE, comprising species such as Li_2_S, Li_*x*_P, and P_2_S_5_. The redox reactions of these interphase species take place synergistically in the lithiation/delithiation process of Al anode, enhancing the overall reversibility of Al anode remarkably. Theoretical calculations show that the as-formed interphase components (Li_2_S, Li_3_P, and LiCl) present a much lower Li^+^ binding energy than that of Li/Al anode, which effectively confines Li^+^ within the anode bulk and prevents uncontrolled Li^+^ migration and anode-SSE interface degradation. Further, this work proposes that the Li^+^ binding energy difference (Δ*E*) between two phases serves as a critical descriptor of interfacial stability. A larger Δ*E* suggests greater Li^+^ preference, inhibited Li^+^ migration and higher interface stability. As a result, the assembled Al anode-based ASSLBs exhibit superior electrochemical performance when paired with high-loading LiNi_0.6_Co_0.2_Mn_0.2_O_2_ (NCM622) cathodes (30 mg cm^−2^) under a stringently low N/P ratio (1.05). They deliver a high specific capacity of 809.25 mAh g^−1^ based on Al with a substantially improved ICE of 84.7% in sharp contrast to 29.85% of baseline pure Al-anode. The stability and fast kinetics are validated in Al-based anode with a high-loading of 30 mg cm^−2^, which shows 54.68% retention after 1000 cycles and retains 1.27 mAh cm^−2^ capacity at a high current density of 7 mA cm^−2^, the robust durability distinguishes the system from analogous benchmarks. These findings highlight the pivotal role of constructing redox-active interphases and provide a highly cost-effective new paradigm for the development of high-performance alloy-conversion type pure metal anodes in ASSLBs.

## Results and discussion

### Design rationale and electrochemical superiority of the Al-LPSC-C anode

**Fig. 1:**
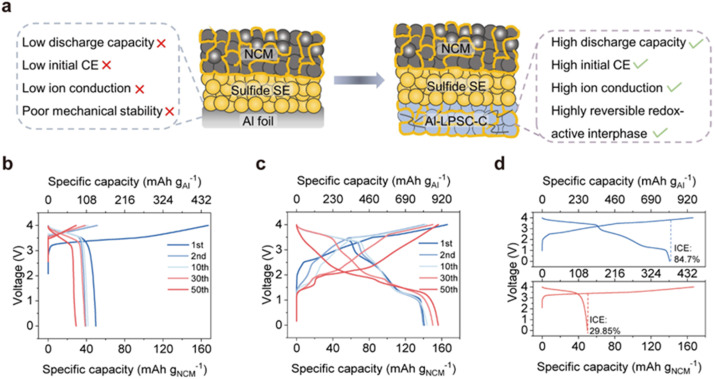
Design principles and electrochemical performance of the Al-LPSC-C composite anode *versus* the Al foil anode. (a) Schematic illustration comparing the Al foil anode (left) with the Al-LPSC-C anode (right). (b) Galvanostatic charge/discharge voltage profiles of the Al foil‖NCM622 full cell at a current density of 0.25 mA cm^−2^. (c) Galvanostatic charge/discharge voltage profiles of the Al-LPSC-C‖NCM622 full cell at a current density of 0.25 mA cm^−2^. (d) A comparative analysis of the ICE for both anodes during the first cycle.

The primary obstacle to deploy pure Al anodes in sulfide-based ASSLBs is the severe chemo-mechanical mismatch between the active material and sulfide SSE. As illustrated in Fig. 1a (left), lithiation of a conventional planar Al foil induces substantial volume expansion, leading to pronounced stress accumulation at the localized planar interface. This mechanical instability triggers a cascade of failure modes, including particle pulverization and detachment of the active material from the ion-conductive network, collectively resulting in poor ionic conduction, low initial coulombic efficiency (ICE), and rapid capacity decay.^29,30^ To address these limitations, we designed a composite anode architecture, denoted Al-LPSC-C, by homogeneously blending Al powder with a sulfide SSE of LPSC and a conductive carbon additive (Fig. 1a, right). In this configuration, LPSC functions not only as an ionic conductor but also as a physical buffer to accommodate volumetric fluctuations and the carbon network ensures continuous electrical conductivity.

To validate this design, full cells were assembled using either an Al foil or the Al-LPSC-C anode paired with a high-loading NCM622 cathode (30 mg cm^−2^). The Al foil anode exhibits severely compromised electrochemical performance at a current density of 0.25 mA cm^−2^, as evidenced by the voltage profiles in Fig. 1b. It shows large polarization and rapid capacity fading over 50 cycles, indicative of kinetic limitations and structural degradation. The first-cycle voltage profiles (Fig. 1d, bottom) further highlight the severity of these issues: the Al foil cell suffers catastrophic irreversible capacity loss, delivering a dismal ICE of only 29.85% and initial discharge capacity is merely 134.78 mAh g^−1^ based on Al. This low efficiency confirms that a substantial fraction of lithium is consumed by the continuous formation of “dead” interfacial regions and isolated metallic Li/Al domains due to contact loss.^29,30^

In stark contrast, the Al-LPSC-C anode exhibits outstanding electrochemical reversibility and stability (Fig. 1c). Remarkably, it delivers a high discharge specific capacity of 809.25 mAh g^−1^ based on Al at a current density of 0.25 mA cm^−2^ (Fig. 1c and S1), along with an initial coulombic efficiency of 84.7% (Fig. 1d, top). Both the first-cycle discharge capacity and ICE are nearly three times higher than those of the aluminum foil counterpart. In extended cycling tests, the Al-LPSC-C anode maintains significantly superior performance compared to the Al foil (Fig. S2), demonstrating that its composite architecture effectively preserves rapid ionic and electronic percolation pathways during cycling while simultaneously mitigating the chemo-mechanical failure modes that afflict conventional Al foils.^31^

Notably, the voltage profile of the Al-LPSC-C anode (Fig. 1c) displays distinct sloping features and plateaus, differing from the typical flat alloying plateaus of pure aluminum. This subtle but critical deviation indicates that the electrochemical reaction mechanism is not solely governed by Li–Al alloying.^32^ We hypothesize that the LPSC component actively participates in the electrochemical reactions, thereby contributing to the enhanced capacity and reversibility. This hypothesis will be rigorously examined in the following sections.

### Electrochemical kinetics of the redox-active interface

**Fig. 2:**
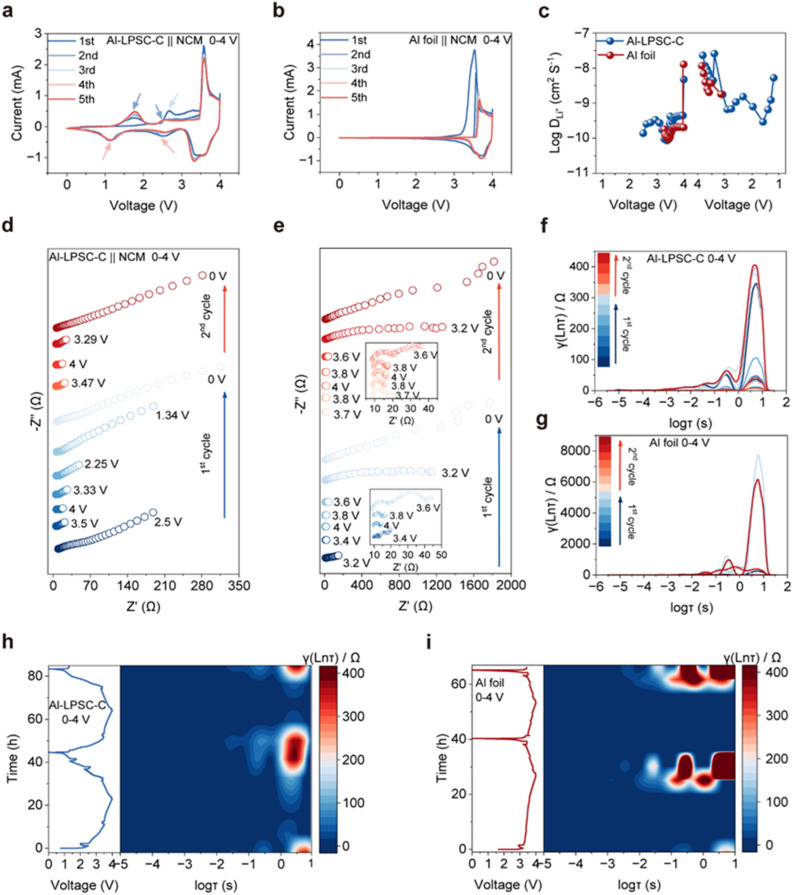
Electrochemical kinetics and interfacial evolution of Al-LPSC-C and Al foil anodes. (a and b) CV curves of the Al-LPSC-C‖NCM622 full cell and the Al foil‖NCM622 full cell. (c) Comparison of lithium-ion diffusion coefficients for both anodes during the first cycle. (d and e) *In situ* EIS Nyquist plots of the Al-LPSC-C and Al foil anodes during the initial two cycles at selected voltage states. (f and g) Corresponding DRT analysis for the Al-LPSC-C and Al foil anodes. (h and i) *Operando* DRT contour maps plotted against voltage and time for the Al-LPSC-C and Al foil anodes.

To elucidate the mechanistic origin of the superior electrochemical performance and verify our hypothesis regarding the active role of LPSC, we conducted a comparative analysis of reaction kinetics and interfacial evolution. Cyclic voltammetry (CV) reveals a clear divergence in the reaction mechanisms of the two anodes. While the Al foil anode exhibits the characteristic, simple redox profiles associated with Li–Al alloying/de-alloying (Fig. 2b),^33,34^ the Al-LPSC-C composite displays a more complex electrochemical signature (Fig. 2a). Specifically, during the initial charge, a distinct oxidation peak emerges at 2.68 V for the Al-LPSC-C anode, which is absent in the Al foil. In subsequent cycles, in addition to the typical Al redox signatures, additional redox couples appear at approximately 1.7 V/2.5 V and 1.1 V/2.5 V (as indicated by the arrows). These features cannot be attributed to Al alone and provide strong evidence that the LPSC component undergoes reversible lithiation and delithiation.^35^ This confirms that LPSC acts not merely as an ionic conductor but also as a redox-active participant, substantially enhancing the overall reversibility and capacity.

This interfacial transformation also profoundly influences ion transport kinetics. Quantified using the Galvanostatic Intermittent Titration Technique (GITT), the Li-ion diffusion coefficient (DLi^+^) of the Al-LPSC-C anode is consistently higher than that of the Al foil across the entire voltage window (Fig. 2c and S3), indicating that incorporation of LPSC significantly improves overall ion transport.^36,37^ Importantly, although LPSC forms a redox-active interphase during cycling, this transformation does not compromise the global reaction kinetics of the anode.

The evolution of this interface under operating conditions was dynamically investigated using *in situ* Electrochemical Impedance Spectroscopy (EIS) combined with Distribution of Relaxation Times (DRT) analysis. Even before cycling, a notable difference in initial impedance is observed between the two anodes, highlighting the substantial improvement in interfacial contact provided by the Al-LPSC-C architecture (Fig. S4). Upon the onset of charging, the impedance of both anodes decreases significantly, corresponding to the formation of conductive Li–Al alloys. However, divergence emerges during discharge: as the potential drops to 3.2 V and below, the Al foil anode experiences a catastrophic increase in impedance, with total resistance rising by an order of magnitude. This sharp increase indicates a rapid loss of active contact area within the anode and at the anode/solid–electrolyte interface, accompanied by the formation of a highly resistive interphase. In contrast, the Al-LPSC-C anode exhibits a markedly different behavior, maintaining a low and stable impedance profile throughout cycling. This stability demonstrates the robustness of the composite architecture and further confirms that the in situ-formed redox-active interphase is highly beneficial.^38^

DRT analysis, which deconvolutes overlapping electrochemical processes based on their time constants (*τ*), provides higher-resolution insights into interfacial dynamics. In the DRT spectrum, relaxation processes with small time constants (10^−6^ to 10^−5^ s) in the high-frequency region correspond to the bulk and grain boundary resistance of the solid-state electrolyte. At lower frequencies, processes with larger *τ* values (10^−4^ to 10^0^ s) are typically associated with interfacial charge transfer at the cathode/SSE and anode/SSE interfaces.^39,40^ Signals at the lowest frequencies (*τ* > 10^0^ s) are generally attributed to diffusion resistance. For the Al foil anode, a broad, pronounced peak appears in the low-frequency region (high *τ*) (Fig. 2g), indicating that sluggish charge transfer across a deteriorating interface is the dominant limitation. In contrast, the Al-LPSC-C anode exhibits well-defined and stable redox peaks (Fig. 2f) that evolve reversibly upon cycling. The appearance of specific time constants in the composite anode aligns with the formation of new interfacial species observed in cyclic voltammetry. *Operando* DRT contour maps further reveal these dynamics: the Al-LPSC-C anode shows coherent and highly reproducible evolution of time constants (Fig. 2h), reminiscent of a regulated “breathing” behavior, whereas the Al foil anode exhibits sporadic emergence and intensification of high-resistance features (Fig. 2i), indicative of progressive interfacial failure. Collectively, these results confirm that the Al-LPSC-C architecture effectively replaces the parasitic interface of pure Al foil with a highly conductive and redox-active interphase.^41^

### Interfacial evolution and reaction mechanism

**Fig. 3:**
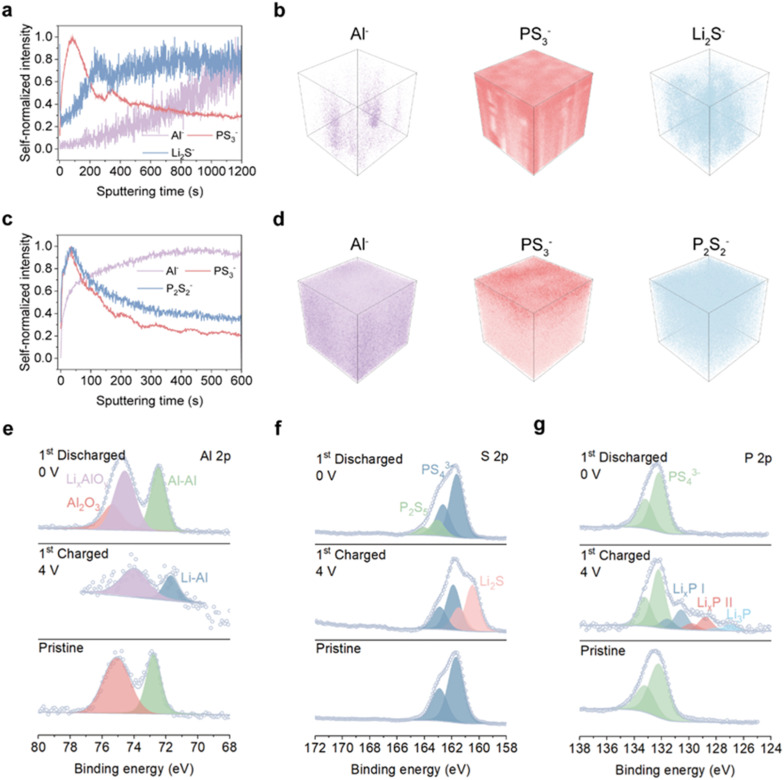
Characterization of the interfacial evolution and reaction mechanism of the Al-LPSC-C anode. (a) Self-normalized ToF-SIMS depth profile at the interface between the Al-LPSC-C anode and the solid-state electrolyte after the initial charge. (b) Corresponding 3D reconstruction of the interface shown in (a). (c) Self-normalized ToF-SIMS depth profile at the interface between the Al-LPSC-C anode and the solid-state electrolyte after the initial discharge. (d) Corresponding 3D reconstruction of the interface shown in (c). (e–g) Al 2p XPS spectra, S 2p XPS spectra and P 2p XPS spectra of the interface between the Al-LPSC-C anode and the SE layer during the initial cycle.

Time-of-flight secondary ion mass spectrometry (TOF-SIMS) was employed to investigate the chemical composition and elemental distribution at the interface between the Al-LPSC-C anode and the SSE, thereby elucidating the evolution of the redox-active interphase during cycling. Fig. 3a, b and S5 present self-normalized depth profiles and three-dimensional (3D) reconstructions of selected secondary ion fragments at the Al-LPSC-C/SSE interface after the first charge. Following charging, significant accumulation of fragments such as Li_2_S^−^ and Li_*x*_P^−^ is observed at the interface. Their depth profiles reveal a uniform distribution independent of sputtering depth, indicating that LPSC actively participates in the lithiation process rather than serving solely as an ionic conductor.^42^ This leads to the formation of uniformly distributed lithiated phases both on the surface and within the bulk of the anode, effectively mitigating stress concentration in Al during alloying. In contrast, after discharge, the signals for Li_2_S^−^ and Li_*x*_P^−^ largely disappear, while characteristic fragments such as P_2_S_2_^−^ emerge instead (Fig. 3c, d and S6). The appearance and transformation of these species correspond to the new redox peaks observed in the cyclic voltammetry profiles. The homogeneous distribution of these phases in both charged and discharged states suggests the formation of a robust, percolating network within the Al-LPSC-C anode during cycling.^38^ This redox-active interface thereby facilitates efficient ion and charge transport throughout the electrode.

X-ray photoelectron spectroscopy (XPS) analysis further elucidates the chemical transformations occurring at the redox-active interface during cycling (Fig. 3e–g, S7 and 8). The Al 2p spectra confirm the reversible Li–Al alloying mechanism: the metallic Al peak (72.7 eV) shifts to a lower binding energy (71.7 eV) upon charging, corresponding to Li–Al alloy formation, and largely reverts to the metallic state after discharge, demonstrating high reversibility.^26^ A residual oxide signal (Li_*x*_AlO_*y*_, 74.3 eV), commonly observed in Al-based anodes and contributing to irreversible Li^+^ consumption, persists throughout cycling and partially accounts for the low initial coulombic efficiency.^17^ More importantly, the dynamic evolution of the S 2p and P 2p spectra provides critical evidence for the reversible redox activity of the LPSC component. In the pristine state, both spectra exhibit characteristic doublets corresponding to P–S bonds. Upon charging, concurrent with Al lithiation, both spectra systematically shift toward lower binding energies, indicating the formation of reduced species. Specifically, the S 2p spectrum reveals satellite peaks at 161.5/160.5 eV assignable to Li_2_S, while the P 2p spectrum indicates the presence of multiple reduced lithium phosphides (Li_*x*_P, 1 ≤ *x* ≤ 3). These reduction products, particularly Li_*x*_P, are known fast ionic conductors, which contributes to the observed reduction in interfacial impedance after charging.^43^ Upon discharge, the lithiated products (Li_2_S and Li_*x*_P) largely disappear, concomitant with delithiation and the formation of higher-valence species such as P_2_S_5_, a transformation further corroborated by Raman spectroscopy (Fig. S9). During subsequent cycles, a dynamic and reversible interconversion is maintained between the reduced (Li_2_S, Li_*x*_P) and oxidized (P_2_S_5_) species (Fig. S7 and 8).^35,44,51^ This reversible compositional transformation corresponds closely with the emergence of the redox peaks observed in cyclic voltammetry (Fig. 2a). Integrating the electrochemical and compositional analyses confirms the multifunctional role of LPSC: it acts not only as an ionic conductor and physical separator preventing direct Al–Al contact, but also as a reversible redox host. The electrochemically active redox interface formed within the Al-LPSC-C anode ensures sustained high ionic conductivity and intimate mechanical contact throughout the electrode bulk and surface, effectively circumventing the poor conductivity and formation of “dead” contacts characteristic of conventional Al-foil anodes.

### Morphological analysis and micro-mechanisms

**Fig. 4:**
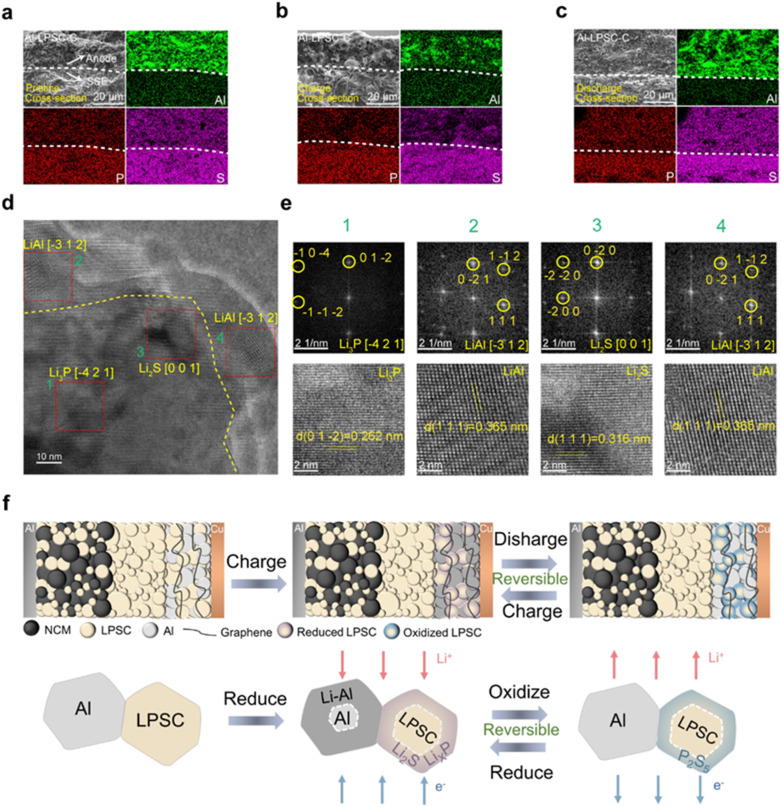
Morphological evolution and atomic-scale phase identification of the Al-LPSC-C anode. (a–c) Cross-sectional SEM images and corresponding EDS elemental mappings of the anode/electrolyte interface at the pristine, charge and discharge states. (d) HRTEM image of the charge anode. (e) The corresponding FFT diffraction pattern in (d) reveals the coexistence of LiAl (alloyed phase) and Li_2_S/Li_3_P (reduction products). (f) Schematic diagram depicting the formation mechanism of the electrochemically active redox interface in the Al-LPSC-C anode during cycling.

To further assess the mechanochemical stability of the anode, cross-sectional scanning electron microscopy (SEM) combined with energy-dispersive X-ray spectroscopy (EDS) mapping was employed to examine its morphological and compositional evolution at different states. In the pristine state, a sharp and well-defined interface is observed between the Al-LPSC-C anode and the SSE layer, with uniform spatial distributions of Al, P, and S elements (Fig. 4a). After full charge and discharge, the cross-section remains free of cracks or additional interphases, and the interface preserves its mechanical integrity (Fig. 4b, c and S11). This behavior contrasts sharply with the severe delamination and particle pulverization observed in the Al foil anode after cycling (Fig. S10). Additionally, the consistently continuous and dense distribution of key elements across the interface provides macroscopic evidence of its chemical stability throughout cycling.

Atomic-scale analysis using transmission electron microscopy (TEM) was performed to elucidate the origin of the exceptional stability in the Al-LPSC-C anode. High-resolution TEM images of the charged anode reveal a representative grain boundary region (Fig. 4d). Fast Fourier transform (FFT) analysis of selected areas exhibiting lattice fringes uncovers the distinctive nanocomposite architecture of the anode (Fig. 4e). The FFT patterns from regions 2 and 4, together with lattice fringes measuring 0.365 nm, are indexed to the (111) planes of the LiAl alloy,^29^ confirming the successful lithiation of Al, consistent with the compositional analyses described earlier. The uniqueness of this composite architecture lies in the encapsulation of LiAl domains by regions identified as Li_2_S (region 3, *d*-spacing = 0.316 nm) and Li_3_P (region 1, *d*-spacing = 0.262 nm).^45,46^ This spatial heterogeneity and close proximity indicate that, during charging, the conversion products of LPSC (Li_2_S and Li_*x*_P) encapsulate the LiAl alloy. This redox-active interface serves a dual function: it provides fast ion-conduction pathways for the alloying reaction and simultaneously accommodates the associated mechanical stress.^47^

Integrating insights from electrochemical testing, compositional analysis, and structural characterization, we propose a dual-active mechanism for the Al-LPSC-C anode, schematically illustrated in Fig. 4f. During charging, Li^+^ migrates to the anode and alloys with Al to form LiAl, a reaction conventionally accompanied by significant volume expansion. Simultaneously, LPSC acts not merely as an ionic conductor but also reacts with Li^+^ to generate reduction products such as Li_2_S and Li_*x*_P. These products encapsulate the LiAl alloy, forming a compliant buffer layer that accommodates volumetric strain while maintaining structural integrity and facilitating ion transport. Upon discharge, Li^+^ is extracted from the LiAl alloy, reverting it to its metallic state, while Li_2_S and Li_*x*_P undergo delithiation to form the oxidized species P_2_S_5_. During subsequent charging, P_2_S_5_ reacts again with Li^+^ to regenerate Li_2_S and Li_*x*_P. This dynamic and reversible transformation establishes an electrochemically active redox interface. Unlike conventional solid electrolytes designed to be inert, the LPSC-derived component actively participates in the redox chemistry, contributing capacity while preserving ionic conduction. The synergistic combination of rapid ion transport, effective accommodation of volume changes, and sustained interfacial contact enables the Al-LPSC-C anode to approach the full theoretical capacity of its Al active material.

### Electrochemical performance and theoretical insights

**Fig. 5:**
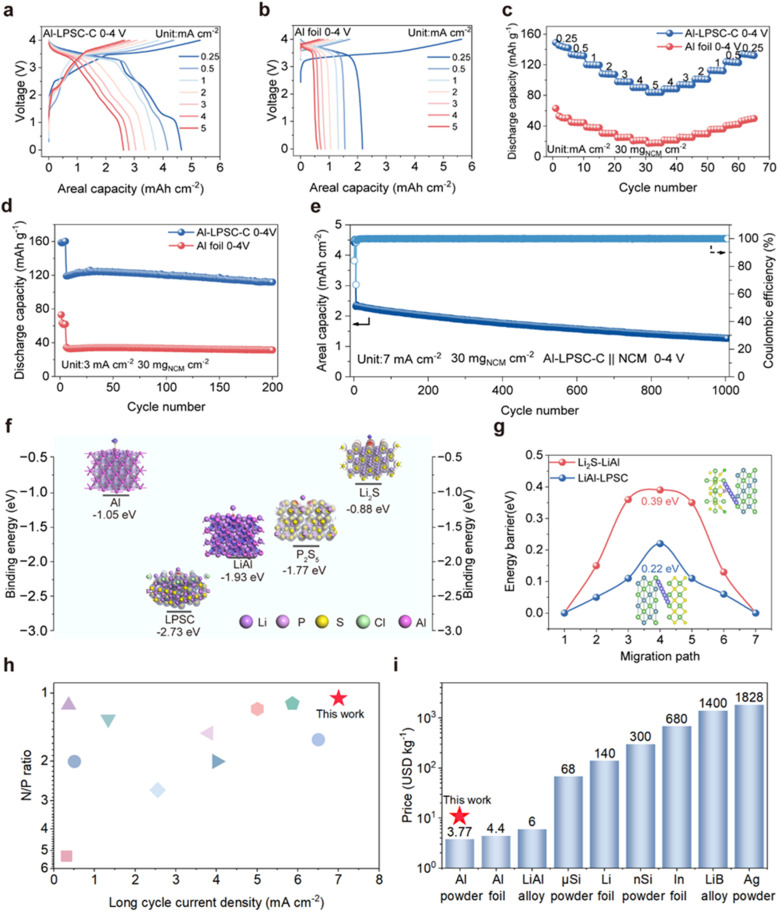
Electrochemical performance and theoretical calculations. (a and b) Galvanostatic charge/discharge profiles of the Al-LPSC-C anode and Al foil anode at various current densities ranging from 0.25 to 5 mA cm^−2^. (c) Rate capability comparison between Al-LPSC-C and Al foil anodes. (d) Cycling performance at a current density of 3 mA cm^−2^. (e) Long cycling stability of the Al-LPSC-C‖NCM full cell under a high current density of 7 mA cm^−2^. (f) Calculation of Li binding energy with various species. (g) Calculation of lithium-ion migration energy at heterogeneous material interfaces. (h) Long cycle performance comparison between the proposed Al anode and state-of-the-art sulfide-based solid-state anodes using full cells with NCM cathodes as a reference. (i) Cost comparison of various anode materials.

Full cells were assembled by pairing the Al-LPSC-C anode with a high-loading NCM622 cathode (30 mg cm^−2^) and subjected to rate capability tests under a stringent N/P ratio of 1.05 to evaluate the kinetic advantages conferred by the redox-active interface. As shown in Fig. 5a, the full cell maintains well-defined voltage plateaus and minimal polarization even at high current densities, indicating that the in situ-formed redox interface enables highly efficient ion transport. The Al-LPSC-C anode delivers high areal capacities of 4.65 and 2.62 mAh cm^−2^ at 0.25 and 5 mA cm^−2^, respectively, with coulombic efficiency exceeding 99% in all cycles following the initial cycle (Fig. S12). By contrast, the Al foil anode exhibits an initial coulombic efficiency below 50%, along with severe voltage hysteresis and rapid capacity fading as the current density increases, with its areal capacity dropping to merely 0.597 mAh cm^−2^ at 5 mA cm^−2^ (Fig. 5b). When normalized to active mass, the Al-LPSC-C anode achieves a specific capacity of 149 mAh g^−1^ at 0.25 mA cm^−2^ and retains 56.4% of this capacity at 5 mA cm^−2^, whereas the Al foil anode delivers only 17.36 mAh g^−1^ under the same high-current condition, unequivocally demonstrating the superiority of the Al-LPSC-C design (Fig. 5c).

The long-term cycling stability of the Al-LPSC-C anode was further evaluated under the same stringent conditions. After 200 cycles at 3 mA cm^−2^, it maintains a specific discharge capacity of 111.9 mAh g^−1^ with 93.94% capacity retention, whereas the Al foil anode, hampered by mechanochemical degradation, retains only 31.33 mAh g^−1^ (Fig. 5d). Notably, at an even higher current density of 7 mA cm^−2^, the Al-LPSC-C anode demonstrates a cycling lifetime exceeding 1000 cycles, with coulombic efficiency remaining close to 100% and an areal capacity of 1.27 mAh cm^−2^ retained after 1000 cycles (Fig. 5e). Even at elevated current densities, stable long-term cycling is maintained (Fig. S13). This exceptional high-rate and long-term cyclability highlights the practical viability of the Al-LPSC-C anode and underscores the pivotal role of the electrochemically active redox interface.

The underlying mechanism for the stable cycling of the Al-LPSC-C anode was investigated through binding energy (BE) calculations between lithium and key constituents at various cycling stages, performed using Materials Studio (MS) (Fig. 5f and S14).^48^ The calculated BEs reveal distinct profiles for the representative phases. In the pristine state, a substantial difference exists between Al (−1.05 eV) and LPSC (−2.73 eV). Upon charging, this difference diminishes between the lithiated product LiAl (−1.93 eV) and LPSC. However, the concurrent formation of redox mediators Li_2_S (−0.88 eV) and Li_*x*_P (−1.17 eV) re-establishes a significant BE differential across the interface. The binding energies of other redox-active components like LiCl are given in SI. After discharge, the BE difference between P_2_S_5_ (−1.77 eV) and LPSC remains modest, while the reversion to metallic Al again increases the overall BE disparity within the system. To further validate the implications of these BE trends, Li^+^ migration energies across interfaces formed between different products were calculated *via* density functional theory (DFT) (Fig. 5g and S15). A consistent correlation is observed between the migration energy trends and the BE differences, indicating a strong link between interfacial energetics and ion transport behavior. For example, the interface between LPSC and LiAl exhibits a relatively small BE difference and a low Li^+^ migration barrier of 0.22 eV, whereas the Li_2_S/LiAl interface shows a larger BE difference and a higher migration barrier of 0.39 eV. A lower Li^+^ migration energy at an interface facilitates Li^+^ shuttling between the two materials, which may promote interfacial self-discharge and compromise stability. Conversely, a higher migration barrier impedes Li^+^ transport in the absence of an external driving force, thereby enhancing interfacial stability. After charging, Li^+^ migration between LiAl and LPSC is thermodynamically favorable; however, the presence of Li_2_S kinetically suppresses this process, preventing immediate Li^+^ shuttling and stabilizing the interface. These observations suggest that the binding energy difference (Δ*E*) between coexisting interfacial phases is a critical descriptor of stability. A pronounced Δ*E* correlates directly with enhanced interfacial robustness, as it generates a strong thermodynamic locking effect among heterogeneous constituents. This energetic contrast promotes robust adhesion, mitigates mechanochemical degradation, and ensures superior interface stability. Consequently, the high stability of the electrochemically active redox interface can be attributed to its large Δ*E*. Characterized by this high Δ*E* landscape, the Al LPSC C anode exhibited low interfacial impedance and excellent cycling stability, the multiphase interface maintains mechanochemical integrity, ultimately enabling long-term cycling with high reversible capacity.

To comprehensively evaluate the practical viability of this design, the performance of the Al-based anode was benchmarked against existing counterparts. As illustrated in Fig. 5h and Table S1, the proposed architecture sustains exceptional long-term reversibility under the coupled extremes of a near-unity N/P ratio and a high current density of ∼7 mA cm^−2^, decisively distinguishing itself among other sulfide-based solid-state anodes.^49^ Crucially, this robust electrochemical resilience is accompanied by a profound economic advantage (Fig. 5i). By utilizing globally abundant Al powder, the active material expense is curtailed to a mere 3.77 USD per kg—nearly two orders of magnitude lower than that of conventional Li (∼140 USD per kg) or In (∼680 USD per kg) foils.^16,20,22,50^ Thus, the compelling synergy of unprecedented cyclic stability under stringent conditions and ultra-low material cost highlights the immense potential of this technological paradigm for scalable, high-energy-density commercial applications.

## Conclusions

In summary, we have successfully developed a highly reversible Al-based composite anode for ASSLBs by engineering a redox-active interface through the electrochemical activation of a Li_5.4_PS_4.4_Cl_1.6_ sulfide electrolyte. Our findings reveal that the *in situ* formed redox-active interface, comprising species such as Li_2_S and Li_*x*_P, not only facilitates rapid Li^+^ transport kinetics but also provides the mechanical resilience necessary to accommodate the volume changes inherent to Al-alloying chemistry. Theoretical insights from DFT calculations established the Li^+^ binding energy difference (Δ*E*) as a critical descriptor for interfacial stability. We demonstrate that the substantial Δ*E* between the generated interphase components creates an energetic landscape that effectively confines Li^+^ within the anode bulk, thereby preventing parasitic ion migration and mitigating interfacial degradation. The large Li^+^ binding energy difference (Δ*E*) across the redox-active interphase translates into a high interfacial migration barrier, which kinetically suppresses self-discharge and preserves interface integrity, while the interphase itself ensures fast Li^+^ transport under operating conditions. This synergetic effect of fast kinetics and structural stability enables the Al anode to overcome its intrinsic limitations, achieving exceptional reversibility even under the most demanding practical conditions. The practical viability of the Al anode is underscored by the remarkable long-term cycling stability of over 1000 cycles achieved under the coupled extremes of high current densities and a stringently low N/P ratio, distinguishing itself among state-of-the-art sulfide-based counterparts. Furthermore, the use of globally abundant Al powder curtails the active material delivering a profound economic advantage over conventional Li or In foils. By highlighting the role of redox-active interface at the Al anode, this work provides a robust strategy for unlocking the potential of high-capacity alloy anodes, paving the way for next-generation, high-energy-density, and safe and highly cost-effective ASSLBs.

## Author contributions

Jiawu Cui: writing – review & editing, writing – original draft, visualization, methodology, formal analysis, data curation, conceptualization. Xiaohui Sun: writing – review & editing, formal analysis. Zhenxin Huang: writing – review & editing, validation. Xianwei Wang: writing – review & editing, formal analysis. Zhen Wang: writing – review & editing, visualization. Zhanhui Jia: writing – review & editing. Chao Wu: writing – review & editing. Kang Yang: formal analysis. Yuping Wu: writing – review & editing. Wei Tang: writing – review & editing, conceptualization, methodology, funding acquisition, formal analysis, resources. Ya-Ling He: writing – review & editing, conceptualization, methodology, funding acquisition, formal analysis.

## Conflicts of interest

There are no conflicts to declare.

## Supplementary Material

SC-017-D6SC03781J-s001

## Data Availability

The data underlying this study are available in the published article and its supplementary information (SI), or available from the authors on request. Supplementary information: detailed experimental procedures for cell fabrication, electrochemical measurements, and theoretical calculations, together with additional data on battery performance, composition, and structural characterization. See DOI: https://doi.org/10.1039/d6sc03781j.
