# Supplementary material for: Engineering a redox-active interface for highly reversible aluminum anode-based practical all-solid-state lithium batteries with ultralow N/P ratio
Source: Chem Sci. 2026 Jun 1;17(27):13433–43. doi: 10.1039/d6sc03781j (PMC13224048; doi:10.1039/d6sc03781j)
Supplement: SC-017-D6SC03781J-s001 [file SC-017-D6SC03781J-s001.pdf]

Supplementary Information (SI) for Chemical Science.
This journal is © The Royal Society of Chemistry 2026

Supplementary Information

Engineering a Redox-Active Interface for Highly Reversible Aluminum Anode-Based Practical All-Solid-State Lithium Batteries with Ultralow N/P Ratio

*Jiawu Cui [1], Xiaohui Sun [2], Zhenxin Huang [1], Xianwei Wang [1], Zhen Wang [1], Zhanhui Jia [3], Chao Wu [1], Kang Yang [4], Yuping Wu [5], Wei Tang *[1], Ya-Ling He *[1]*

[1] School of Chemical Engineering and Technology, National Innovation Platform (Center) for Industry-Education In-tegration of Energy Storage Technology, State Key Laboratory of Fluorine & Nitrogen Chemicals, Xi'an Jiaotong University, Xi'an 710049, P. R. China.

[2] State Key Lab Space Power Sources, Shanghai Institute Space Power Sources, Shanghai 200245, P. R. China.

[3] School of Materials Science and Engineering, Xi'an Jiaotong University, Xi'an, 710049, P. R. China.

[4] Top−Energy Digital Manufacturing Technologies (Xi'an) Co., Ltd. No. 2090, Hangtuo Road, Xi'an, 710100, P. R. China.

[5] School of Energy and Environment, Southeast University, Nanjing, 210096, P. R. China.

*Corresponding Author

Ya-Ling He : Email: yalinghe@mail.xjtu.edu.cn; Wei Tang : Email: tangw2018@mail.xjtu.edu.cn

Materials and methods

Material Preparation: The composite anode was prepared by ball-milling Al powder (Yamei Nano), $Li_{5.4}PS_{4.4}Cl_{1.6}$ (ZhongkeGuneng New Energy Technology Co., Ltd) and graphene (XFNANO) in a mass ratio of 4:5:1 for 5 hours at 400 rpm within an argon-filled glove box. Al foil (ommercial grade, MTI-kejing) was directly into circular discs with a diameter of 10 mm for use. The NCM622 composite cathode was fabricated by manually grinding the NCM622 (Canrd) with $Li_{5.4}PS_{4.4}Cl_{1.6}$ at a mass ratio of 7:3 for 20 minutes inside an argon-filled glove box.

Battery Assembling: All batteries are assembled in an argon-filled glovebox with H_2O and O_2 concentrations of 0.1 ppm. To fabricate the Al-LPSC-C || NCM622 cells, 100 mg of LPSC was added to a PEEK die with a diameter of 10 mm, and then 10 mg of Al-LPSC-C was cast on one side of the SE. Subsequently, 33.6 mg of the prepared NCM622 composite cathode was evenly spread onto the opposite face of the SE pellet, followed by cold-pressing under a pressure of 3 tons. The assembly of the Al foil || NCM622 cells followed the same process as described above, except that the 10 mg of Al-LPSC-C on the one side was replaced with Al foil (10 mg).

Electrochemical Tests: The Al-LPSC-C || NCM622 cell was tested within the voltage range of 0−4 V at a stacking pressure of 125 MPa. CV measurements were performed from 0 to 4V at 0.08 mV s^{-1}. In the EIS tests of the cell, the test frequencies were in the range of 0.1 Hz to 10 MHz with an AC amplitude of 10 mV. The test conditions for Al foil || NCM622 cell are identical to those for Al-LPSC-C || NCM622 cell. All cells were tested at 60 °C and at NEWARE tester.

Characterization. EIS and CV tests were conducted on a DONGHUA chemical workstation. A scanning electron microscope (TESCAN MAIA3 LMH) was used to examine the morphology of the cells. X-ray photoelectron spectroscopy (XPS, AXISULTRADLD-600W) was performed to further characterize the composition of the interface. TOF-SIMS characterization was performed using a TOF-SIMS 5−100 spectrometer (IONTOF M6). The sputtering times were set at 600 s and 1200 s, with a sputtering area of 50 × 50 μm^2. The TEM characterization was performed using a

Lorenz Transmission Electron Microscope (Talos F200X). All of the testing procedures were strictly insulated from air.

Calculations: The binding energy calculations were performed using the CASTEP module implemented in the Materials Studio 2023 software package based on density functional theory (DFT). The electron–ion interactions were described using the pseudopotential approach. The exchange–correlation energy was treated within the generalized gradient approximation (GGA) using the Perdew – Burke – Ernzerhof (PBE) functional. The interactions between valence electrons and ionic cores were represented by ultrasoft pseudopotentials. The structural models of Al, Li, and P_2S_5 were constructed from the built-in crystal database of Materials Studio and were fully optimized prior to further calculations. During structural optimization, the atomic positions, lattice parameters, and cell volume were fully relaxed until the energy and force convergence criteria were satisfied. To ensure the reliability and convergence of the calculations, the plane-wave energy cutoff was set to 500 eV. For the binding energy calculations, Li^+ ions were placed at representative high-symmetry sites or energetically favorable stable configurations within the corresponding systems, followed by a full geometric optimization to obtain the most stable structures. To eliminate artificial interactions arising from periodic boundary conditions, a vacuum layer of at least 10 Å was introduced for non-three-dimensionally periodic systems.

The binding energy between Li^+ and the different systems was calculated according to the following equation:

$$E_{int} = E_{AB} - E_A - E_B$$

All Li^+ migration energy calculations were carried out for the material in the framework of Density Functional Theory (DFT) using the Vienna Ab initio Simulation Package (VASP 6.3.0). The generalised gradient approximation (GGA) of the Perdew-Burke-Ernzerhof (PBE) function was used to describe the exchange-correlation energy. The projected augmented wave (PAW) method and pseudopotentials were used to describe the interactions between valence electrons and ions. To ensure the efficiency of the computational results and parallel computing. A 3*3*1 k-point grid under Monkhorst-Pack is used in the optimisation process and 450 eV truncation energy is set. The lattice

parameters and ionic positions of all crystals were fully relaxed, and the convergence criteria for the total energy of all relaxed atoms and the final force were 10-6 eV and 0.03 eV/Å, respectively.

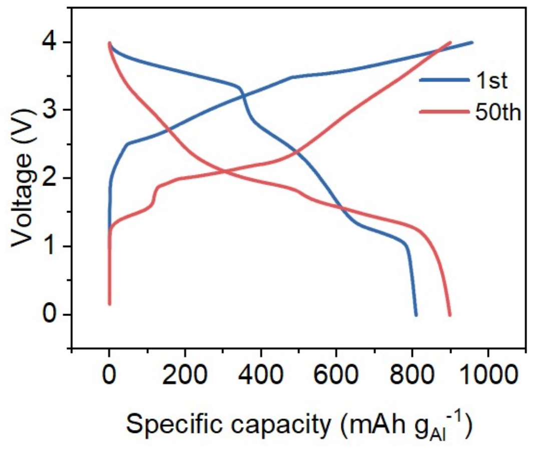

Supplementary Figure 1. Al-LPSC-C || NCM622 full cell charge/discharge capacity with Al as active material at a current density of 0.25 mA cm^{-2}.

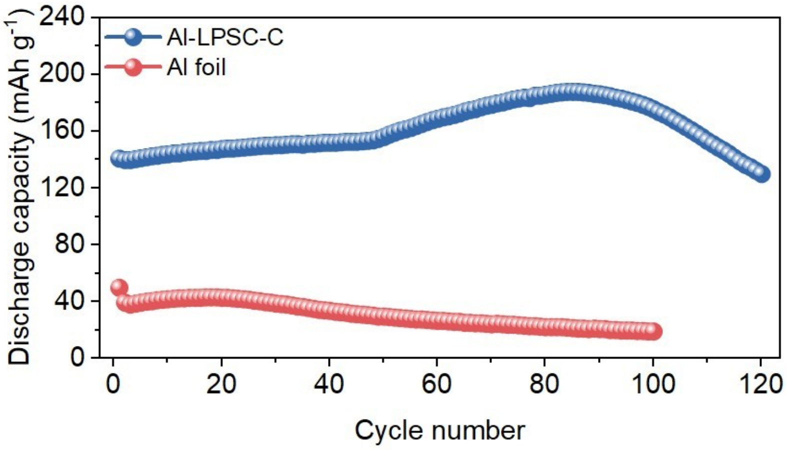

Supplementary Figure 2. A comparative evaluation of the long-term cycling performance for both anodes was conducted at a current density of 0.25 mA cm^{-2}.

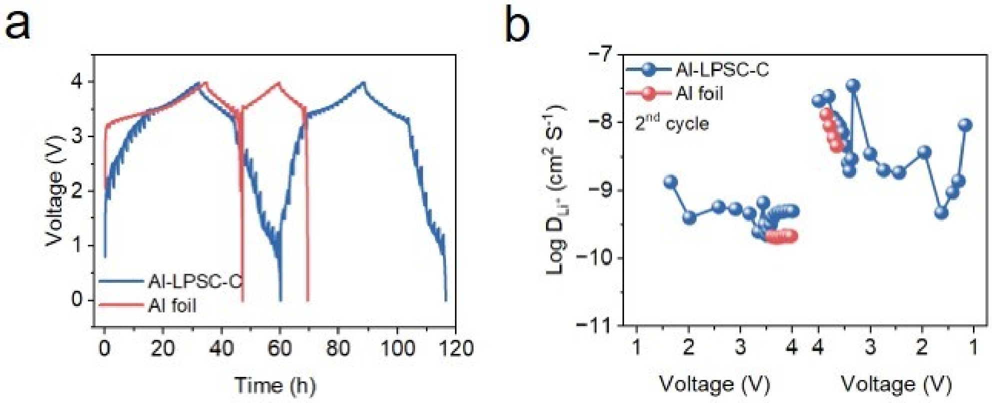

Supplementary Figure 3. (a) GITT voltage profiles of both anodes for the initial two cycles. (b) Comparative analysis of lithium-ion transport coefficients during the second cycle.

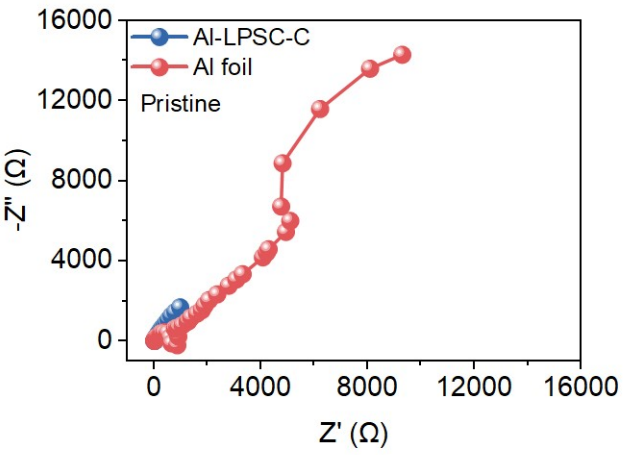

Supplementary Figure 4. Comparative analysis of the initial impedance for both anodes.

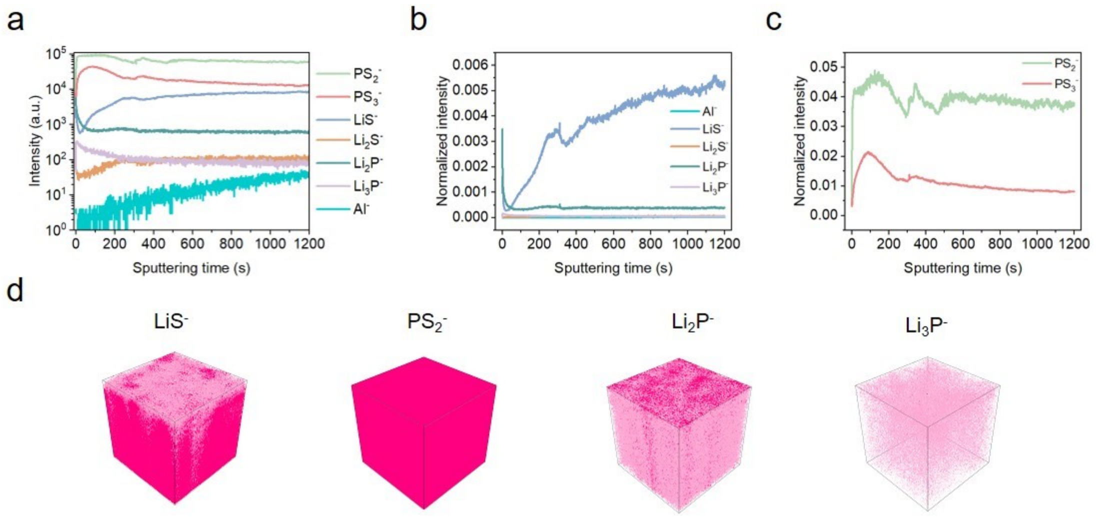

Supplementary Figure 5. (a) Depth profile of the raw TOF-SIMS signal intensity at the interface between the Al-LPSC-C anode and the SE layer after the initial charge. (b, c) Depth profiles of selected secondary-ion fragments from (a) after normalization to total ion counts. (d) Corresponding 3D reconstruction image.

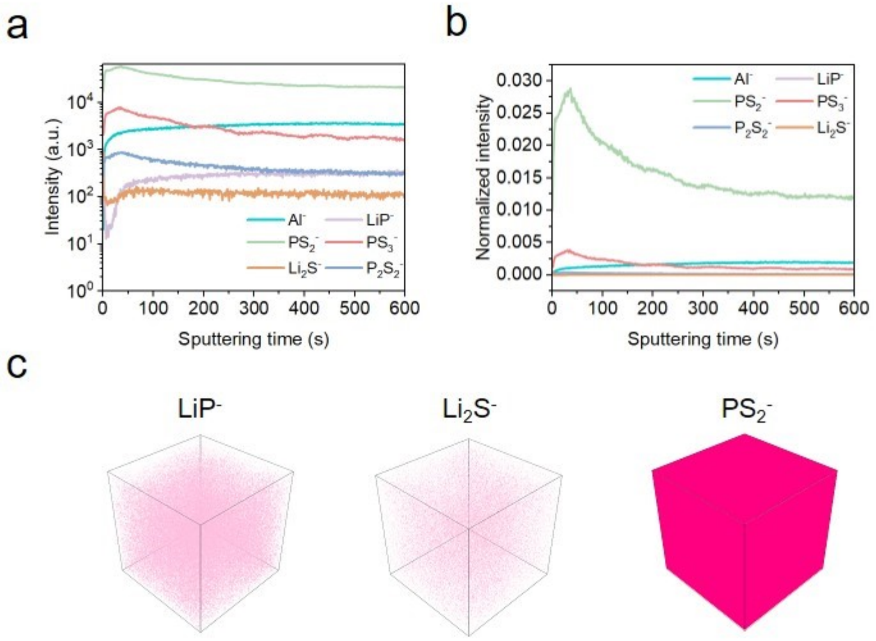

Supplementary Figure 6. (a) Depth profile of the raw TOF-SIMS signal intensity at the interface between the Al-LPSC-C anode and the SE layer after the initial cycle. (b) Depth profiles of selected secondary-ion fragments from (a) after normalization to total ion counts. (c) Corresponding 3D reconstruction image.

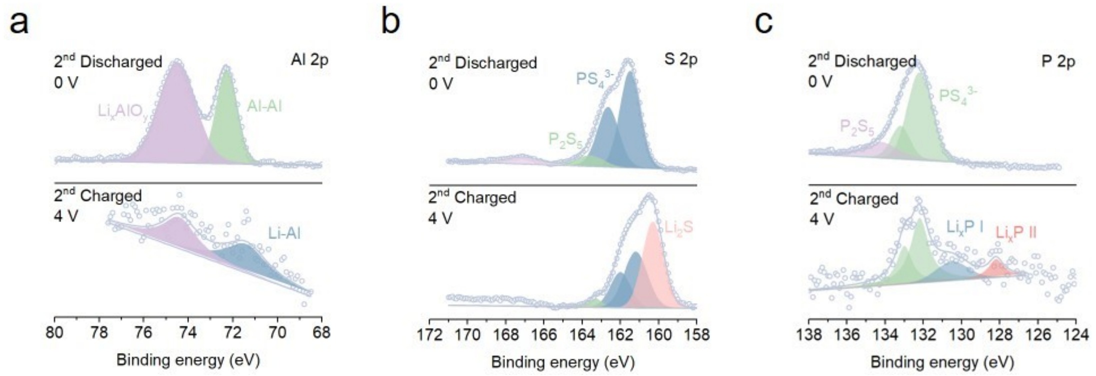

Supplementary Figure 7. (a) Al 2p XPS spectra, (b) S 2p XPS spectra, and (c) P 2p XPS spectra of the interface between the Al-LPSC-C anode and the SE layer during the second cycle.

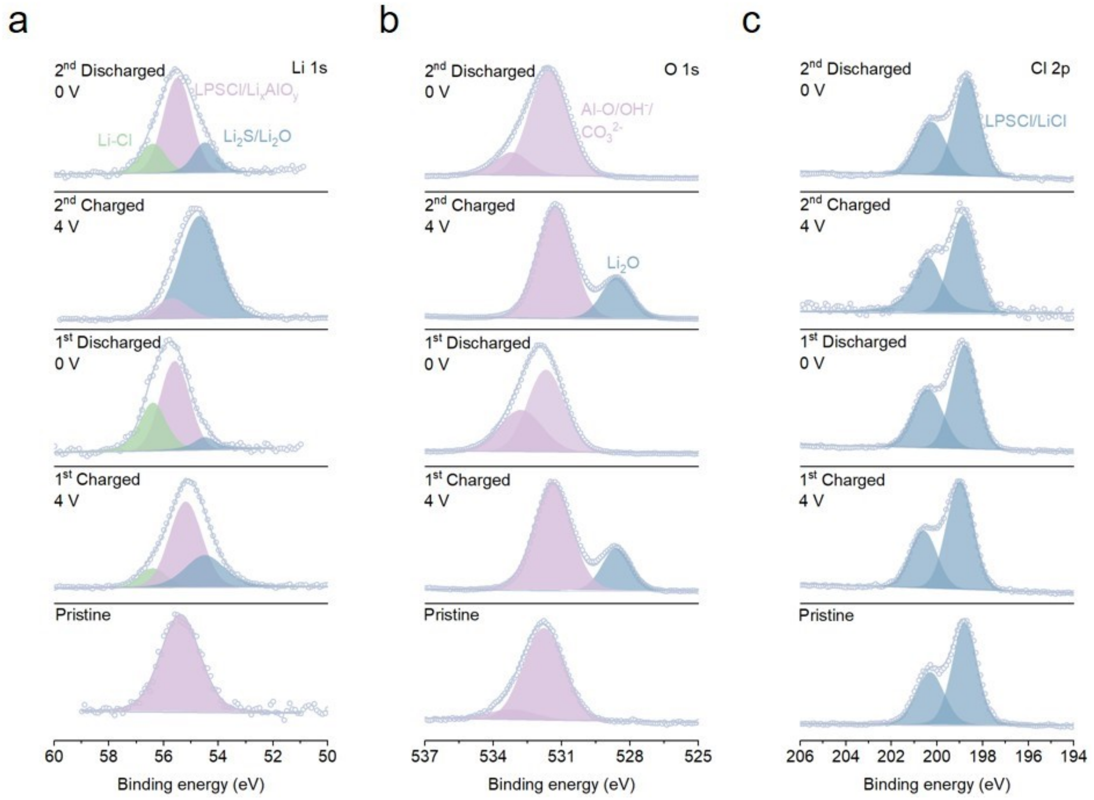

Supplementary Figure 8. (a) Li 1s XPS spectra, (b) O 1s XPS spectra, and (c) Cl 2p XPS spectra of the interface between the Al-LPSC-C anode and the SE layer during the initial second cycle.

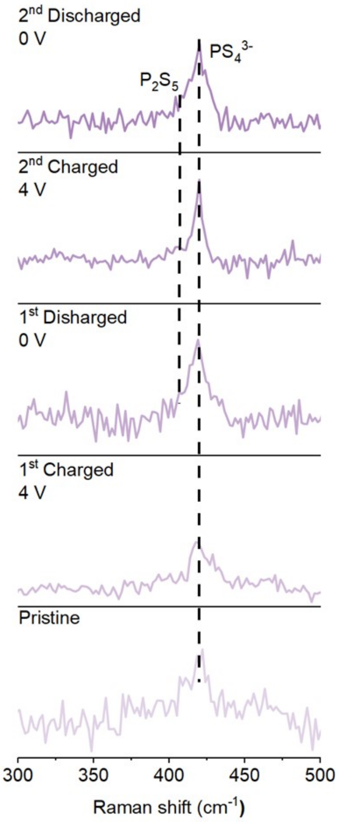

Supplementary Figure 9. Raman spectra of the interface between the Al-LPSC-C anode and the SE layer during the initial second cycle.

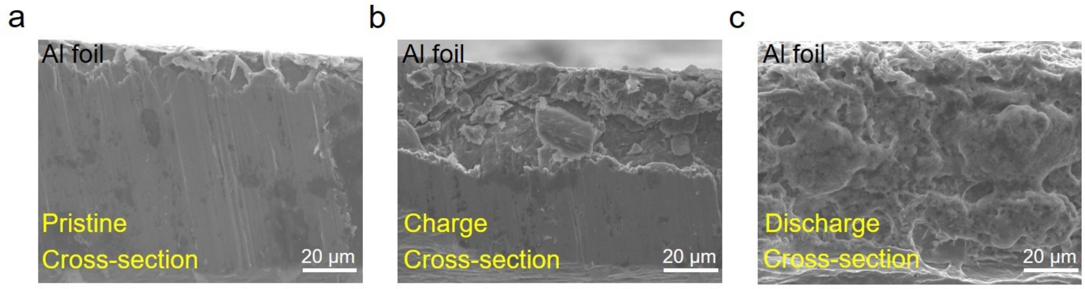

Supplementary Figure 10. Comparative cross-sectional imaging of the Al foil anode during the first cycle.

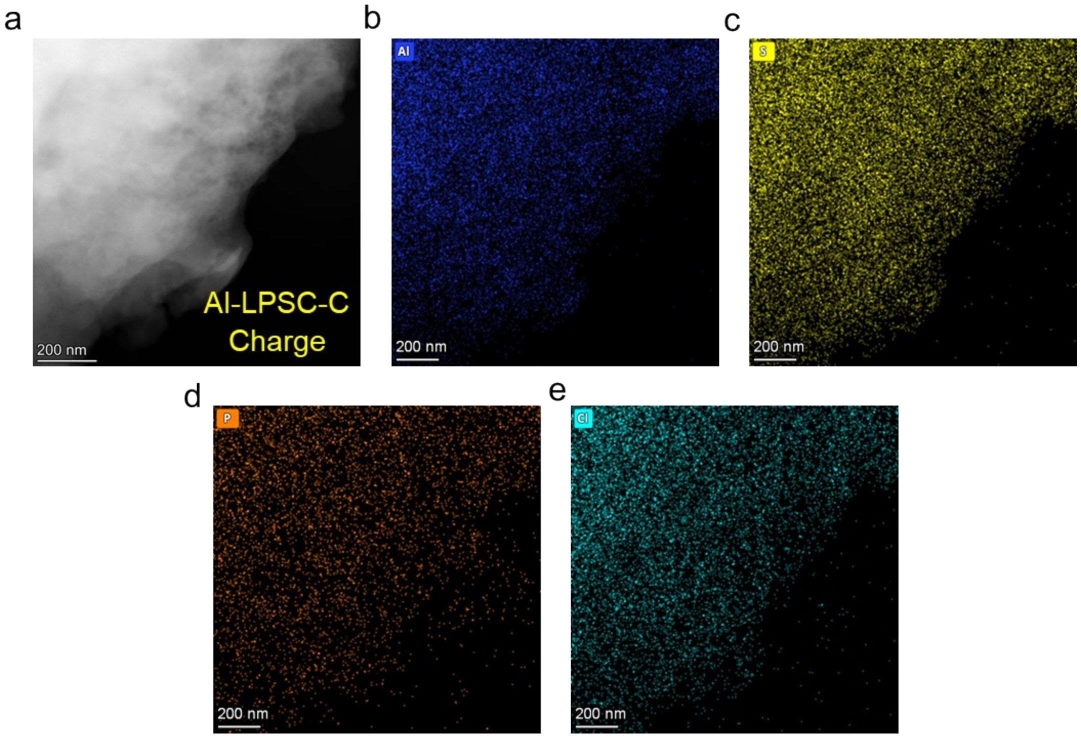

Supplementary Figure 11. EDS analysis of Al-LPSC-C anode after charging in HRTEM mode.

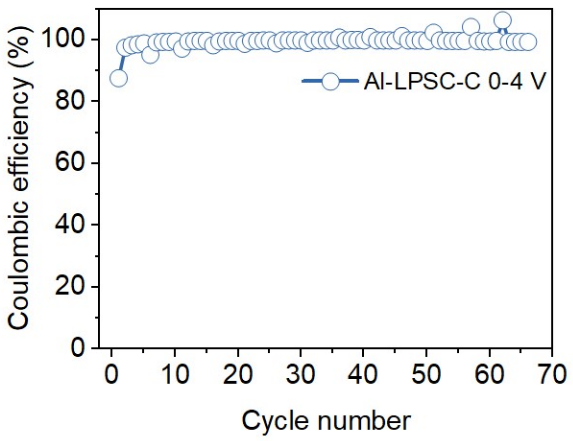

Supplementary Figure 12. The CE profile of the Al-LPSC-C anode during rate capability testing.

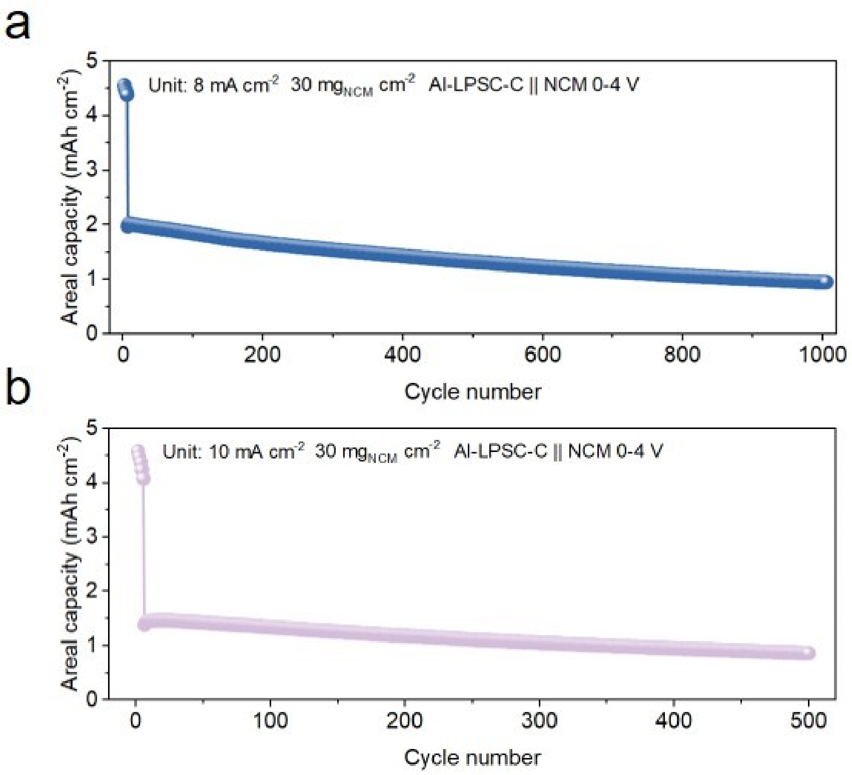

Supplementary Figure 13. Long cycling performance of the Al-LPSC-C || NCM full cell at 8 mA cm^{-2} (a) and 10 mA cm^{-2} (b).

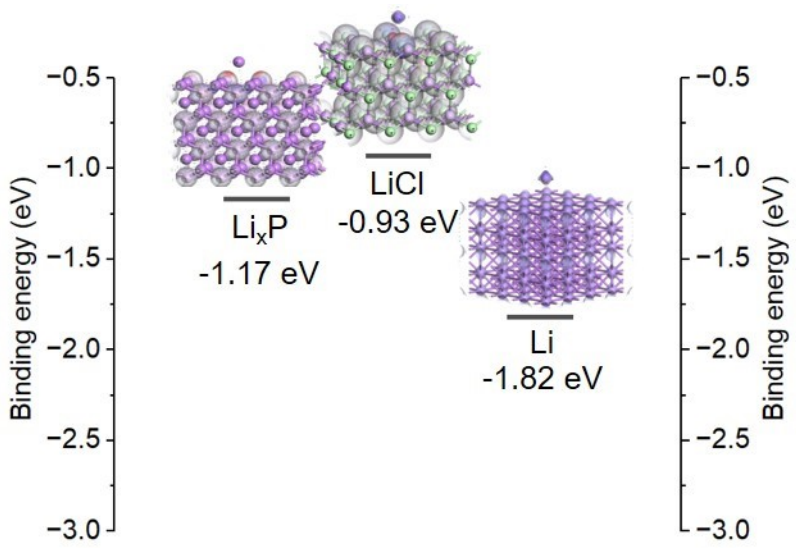

Supplementary Figure 14. Calculation of Binding energies for other substances during cycling.

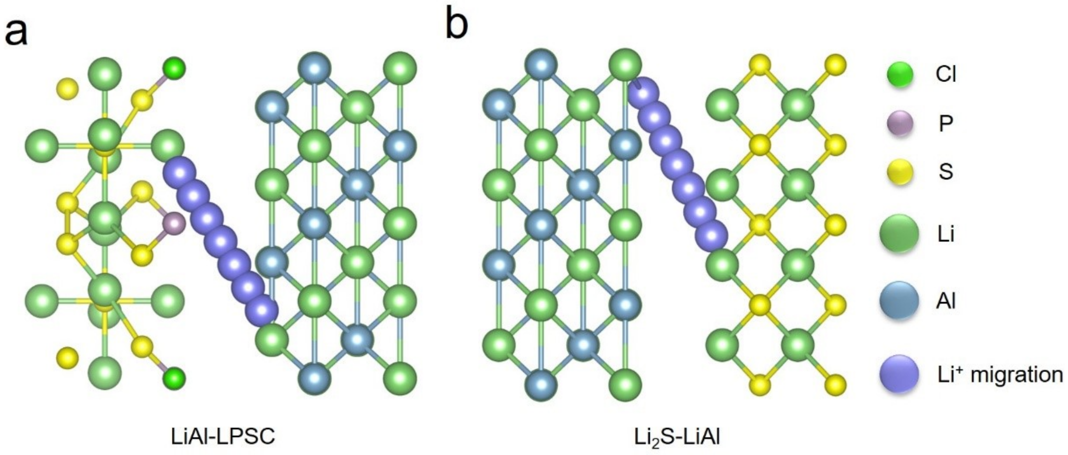

Supplementary Figure 15. Construction of a lithium-ion migration model at the interface and its migration pathway diagram.

Supplementary Table 1. Summary of the recently reported anode performance in all-solid-state sulfide batteries.

Anode	N/P ratio	Areal capacity	Number of long cycle	Long cycle current density	Cathode	ICE	References
Al powder	1.05	5.3 mAh cm^{-2}	1000	7 mA cm^{-2}	30 mg cm^{-2} NCM622	84.7%	This work
Al foil	1.6	5.8 mAh cm^{-2}	200	6.5 mA cm^{-2}	31 mg cm^{-2} NCM622	64%	[1]
Al-In foil	1.6	5.8 mAh cm^{-2}	200	6.5 mA cm^{-2}	31 mg cm^{-2} NCM622	85%	[1]
$Li_{0.5}Al$	2	5 mAh cm^{-2}	2000	4 mA cm^{-2}	25.1 mg cm^{-2} NCM622	76%	[2]
LiAl-d	2.68	4 mAh cm^{-2}	770	2.55 mA cm^{-2}	12.74 mg cm^{-2} NCM811	79.7%	[3]
LiAl-p	2.68	4 mAh cm^{-2}	1800	2.55 mA cm^{-2}	12.74 mg cm^{-2} NCM811	79.6%	[3]
$Li_{0.8}Al$	1.125	1.79mAh cm^{-2}	200	0.36 mA cm^{-2}	1.07mg cm^{-2} S	91.7%	[4]
μSi	1.17	5 mAh cm^{-2}	500	5 mA cm^{-2}	25 mg cm^{-2} NCM811	80.3%	[5]
Li-Ag	12.1	2.6 mAh cm^{-2}	140	0.47 mA cm^{-2}	13 mg cm^{-2} NCM622	88%	[6]
Li-In	1.5	4 mAh cm^{-2}	800	3.8 mA cm^{-2}	35.6 mg cm^{-2} NCM622	86%	[7]
nSi	1.3	4 mAh cm^{-2}	1000	1.33 mA cm^{-2}	20 mg cm^{-2} NCM811	83.9%	[8]
Li-Si-HC	1.11	5.86 mAh cm^{-2}	5000	5.86 mA cm^{-2}	18.66 mg cm^{-2} NCM811	83%	[9]
LiB-AgC	5.3	1.5 mAh cm^{-2}	60	0.32 mA cm^{-2}	1.3mg cm^{-2} S	99.9%	[10]
Ni-Ag-C	2	3.1 mAh cm^{-2}	100	0.5 mA cm^{-2}	17 mg cm^{-2} NCM811	67.7%	[11]
$Li-LiC_6-Li_{22}Sn_5$	8	1.25 mAh cm^{-2}	100	0.25 mA cm^{-2}	6.24 mg cm^{-2} NCM90	93%	[12]

References

[1]. Liu, Y.; Wang, C.; Yoon, S. G.; Han, S. Y.; Lewis, J. A.; Prakash, D.; Klein, E. J.; Chen, T.; Kang, D. H.; Majumdar, D.; Gopalaswamy, R.; McDowell, M. T., Aluminum foil negative electrodes with multiphase microstructure for all-solid-state Li-ion batteries. *Nat. Commun.* **2023,** *14* (1), 3975.

[2]. Jeon, Y.; Lee, D. J.; Zheng, H.; Behara, S. S.; Lee, J. P.; Wu, J.; Li, F.; Tang, W.; Zhang, L.; Chen, Y. T.; Xu, D.; Kim, J.; Song, M. S.; Van der Ven, A.; He, K.; Chen, Z., Lithium diffusion-controlled Li-Al alloy negative electrode for all-solid-state battery. *Nat. Commun* **2025,** *16* (1), 9629.

[3]. Zhu, J.; Luo, J.; Li, J.; Huang, S.; Geng, H.; Chen, Z.; Jia, L.; Fu, Y.; Zhang, X.; Zhuang, X., A Porous Li-Al Alloy Anode toward High-Performance Sulfide-Based All-Solid-State Lithium Batteries. *Adv. Mater.* **2024,** *36* (39), e2407128.

[4]. Hui Pan, M. Z., Zhu Cheng, Heyang Jiang, Jingui Yang, Pengfei Wang, Ping He*, Haoshen Zhou, Carbon-free and binder-free Li-Al alloy anode enabling an all-solid-state Li-S battery with high energy and stability. *Sci.Adv* **2022**, 8, eabn4372.

[5]. Tan, D. H. S.; Chen, Y.-T.; Yang, H.; Bao, W.; Sreenarayanan, B.; Doux, J.-M.; Li, W.; Lu, B.; Ham, S.-Y.; Sayahpour, B.; Scharf, J.; Wu, E. A.; Deysher, G.; Han, H. E.; Hah, H. J.; Jeong, H.; Lee, J. B.; Chen, Z.; Meng, Y. S., Carbon-free high-loading silicon anodes enabled by sulfide solid electrolytes. *Science* **2021,** *373* (6562), 1494-1499.

[6]. Choi, H. J.; Kang, D. W.; Park, J.-W.; Park, J.-H.; Lee, Y.-J.; Ha, Y.-C.; Lee, S.-M.; Yoon, S. Y.; Kim, B. G., In Situ Formed Ag-Li Intermetallic Layer for Stable Cycling of All-Solid-State Lithium Batteries. *Adv. Sci.* **2022,** *9* (1), 2103826.

[7]. Luo, S.; Wang, Z.; Li, X.; Liu, X.; Wang, H.; Ma, W.; Zhang, L.; Zhu, L.; Zhang, X., Growth of lithium-indium dendrites in all-solid-state lithium-based batteries with sulfide electrolytes. *Nat. Commun.* **2021,** *12* (1), 6968.

[8]. Cao, D.; Sun, X.; Li, Y.; Anderson, A.; Lu, W.; Zhu, H., Long-Cycling Sulfide-Based All-Solid-State Batteries Enabled by Electrochemo-Mechanically Stable Electrodes. *Adv. Mater.* **2022,** *34* (24), 2200401.

[9]. Yan, W.; Mu, Z.; Wang, Z.; Huang, Y.; Wu, D.; Lu, P.; Lu, J.; Xu, J.; Wu, Y.; Ma, T.; Yang, M.; Zhu, X.; Xia, Y.; Shi, S.; Chen, L.; Li, H.; Wu, F., Hard-carbon-stabilized Li–Si anodes for

high-performance all-solid-state Li-ion batteries. *Nat. Energy* **2023,** *8* (8), 800-813.

[10]. Chen, Z.; Liang, Z.; Zhong, H.; Su, Y.; Wang, K.; Yang, Y., Bulk/Interfacial Synergetic Approaches Enable the Stable Anode for High Energy Density All-Solid-State Lithium–Sulfur Batteries. *ACS Energy Lett.* **2022,** *7* (8), 2761-2770.

[11]. Park, S. H.; Jun, D.; Lee, G. H.; Lee, S. G.; Jung, J. E.; Bae, K. Y.; Son, S.; Lee, Y. J., Designing 3D Anode Based on Pore-Size-Dependent Li Deposition Behavior for Reversible Li-Free All-Solid-State Batteries. *Adv. Sci.* **2022,** *9* (28), 2203130.

[12]. Li, J.; Su, H.; Jiang, Z.; Zhong, Y.; Wang, X.; Gu, C.; Xia, X.; Tu, J., Domain-limited laminar lithium deposition behavior mediated by the design of hybrid anode for sulfide-based all-solid-state batteries. *Acta Mater.* **2023,** *244*, 118592.
